# The Antibacterial Activity of Ta-doped ZnO Nanoparticles

**DOI:** 10.1186/s11671-015-1047-4

**Published:** 2015-08-21

**Authors:** Bing-Lei Guo, Ping Han, Li-Chuan Guo, Yan-Qiang Cao, Ai-Dong Li, Ji-Zhou Kong, Hai-Fa Zhai, Di Wu

**Affiliations:** National Laboratory of Solid State Microstructures, Materials Science and Engineering Department, College of Engineering and Applied Sciences, Collaborative Innovation Center of Advanced Microstructures, Nanjing University, Nanjing, 210093 People’s Republic of China; College of Life Science, Nanjing University, Nanjing, 210093 People’s Republic of China

**Keywords:** Ta-doped ZnO, Photocatalytical inactivation, Minimum inhibitory concentration, Antibacterial activity

## Abstract

A novel photocatalyst of Ta-doped ZnO nanoparticles was prepared by a modified Pechini-type method. The antimicrobial study of Ta-doped ZnO nanoparticles on several bacteria of Gram-positive *Bacillus subtilis* (*B. subtilis*) and *Staphylococcus aureus* (*S. aureus*) and Gram-negative *Escherichia coli* (*E. coli*) and *Pseudomonas aeruginosa* (*P. aeruginosa*) were performed using a standard microbial method. The Ta-doping concentration effect on the minimum inhibitory concentration (MIC) of various bacteria under dark ambient has been evaluated. The photocatalytical inactivation of Ta-doped ZnO nanoparticles under visible light irradiation was examined. The MIC results indicate that the incorporation of Ta^5+^ ions into ZnO significantly improve the bacteriostasis effect of ZnO nanoparticles on *E. coli*, *S. aureus*, and *B. subtilis* in the absence of light. Compared to MIC results without light irradiation, Ta-doped ZnO and pure ZnO nanoparticles show much stronger bactericidal efficacy on *P. aeruginosa*, *E. coli*, and *S. aureus* under visible light illumination. The possible antimicrobial mechanisms in Ta-doped ZnO systems under visible light and dark conditions were also proposed. Ta-doped ZnO nanoparticles exhibit more effective bactericidal efficacy than pure ZnO in dark ambient, which can be attributed to the synergistic effect of enhanced surface bioactivity and increased electrostatic force due to the incorporation of Ta^5+^ ions into ZnO. Based on the antibacterial tests, 5 % Ta-doped ZnO is a more effective antimicrobial agent than pure ZnO.

## Background

In recent years, ZnO has received increasing attention, owing to its unique optical, electrical, and chemical properties [1]. Among these properties, degradation of pollutants catalyzed by ZnO has been studied widely so far [2–4]. Furthermore, ZnO appears to strongly resist microorganisms [5, 6]. Some work on the considerable antibacterial activity of bulk and nanosized ZnO materials has been reported [7–10].

Antibacterial agents are of relevance to a number of industrial sectors including environmental, water disposal, food, synthetic textiles, packaging, healthcare, medical care, as well as construction and decoration. They can be classified into two types, organic and inorganic ones. Most organic antibacterial agents are sensitive to temperature or pressure [11], while inorganic materials such as metal and metal oxide [12] have received more recognition over the past decade due to superior durability, less toxicity, greater selectivity, and heat resistance [13, 14]. TiO_2_ and ZnO semiconductors have been extensively studied as antimicrobial agents due to their photocatalytic activity under UV light [15, 16].

ZnO, as a semiconductor with a wide band gap (3.3 eV), is abundant in nature and environmentally friendly with low price. ZnO nanoparticles have been reported to exhibit strong antibacterial activities on a broad spectrum of bacteria [7–11]. A comparative study showed that for *Bacillus subtilis* and *Escherichia coli*, biocidal activity generally increased from SiO_2_ to TiO_2_ to ZnO [8]. ZnO has higher bactericidal efficacy on *B. subtilis* than on *E. coli* [5]. The minimum inhibitory concentration (MIC) was dependent on the ZnO particle size, ranging from 2000 to 12,500 ppm for *B. subtilis* and 50,000 to 100,000 ppm for *E. coli* [6]. However, contradictory results have been reported on the impact of particle size on the antibacterial activity of ZnO [17, 18]. Up to now, the antibacterial mechanism of ZnO is still under investigation and not well understood [19]. Several possible mechanisms, including the penetration of the cell envelope and disorganization of bacterial membrane [10], the photocatalytically generated reactive oxygen species (ROS) on oxide surface [11], and Zn^2+^ ion binding to the membranes of microorganisms [20], have been suggested.

ZnO has high UV absorption efficiency and good transparency to visible light. In order to improve its photocatalytic properties, some metal ions or doping nitrogen have been added into ZnO to narrow or split the band gap and enhance interfacial electron-transfer rate [21–23]. Some transition metal ions, e.g., Co^2+^ [24], Mn^2+^ [22], Ti^4+^ [25], La^3+^ [26], and Fe^3+^ [27], have been doped into ZnO. At present, most research focused on the influence of transition metal ions on the photocatalytic efficiency rather than the antimicrobial activity [22, 23].

Our group has first prepared Ta-doped ZnO nanopowders by a modified Pechini-type method and deeply investigated the photoinduced degradation of organic dye of methylene blue using Ta-doped ZnO with various Ta contents under visible light irradiation [28, 29]. In this work, we performed the antibacterial study of Ta-doped ZnO nanoparticles on several bacteria of Gram-positive *Bacillus subtilis* (*B. subtilis*) and *Staphylococcus aureus* (*S. aureus*) and Gram-negative *Escherichia coli* (*E. coli*) and *Pseudomonas aeruginosa* (*P. aeruginosa*) using a standard microbial method. The Ta concentration effect on the MIC of various bacteria has been evaluated. The antimicrobial activity of Ta-doped ZnO under visible light and dark conditions was examined and compared. The possible antimicrobial mechanism in Ta-doped ZnO systems was also proposed.

## Methods

### Preparation of Ta-doped ZnO Nanoparticles

A photocatalyst of Ta-doped ZnO with various Ta contents of 1, 3, and 5 % (mol. percentage) and pure ZnO was prepared by a modified Pechini-type method [27]. All chemicals were analytical grade and used without further purification. Zinc nitrate Zn(NO_3_)_2_·6H_2_O and the water-soluble peroxo-citrato-tantalum were chosen as precursors of Zn and Ta, respectively. The synthesis of home-made water-soluble peroxo-citrato-tantalum has been described elsewhere in details [30]. Deionized water was used as a dispersing agent in all the experiments.

Citric acid (CA) as chelating agent was dissolved into 50 ml water with 2.98 g Zn(NO_3_)_2_·6H_2_O (in 2:1 molar ratio with respect to the zinc nitrate). Ethylene glycol (EG) as cross-linking additive was added into the aqueous Ta precursor solution at the molar ratio of EG to CA of 5:1. As surfactant and catalyst, 1 g polyvinyl pyrrolidone (PVP, average mol. wt. 100,000) and 0.5 ml HNO_3_ were added to the mixture of solutions containing Zn and Ta, respectively. Hydroxypropyl cellulose (HPC, average mol. wt. 100 000, Aldrich) with a concentration 3.5 × 10^−3^ g/ml and 1 g acetylacetone were put into the above solution as steric dispersant and stabilizer, respectively. A colorless transparent sol was obtained and subsequently baked at 140 °C for 12 h for polyesterification. The resultant dark gray glassy resin finally underwent a two-step heat treatment to yield the final products: firstly, pyrolysis at 400 °C for 2 h and then annealing at 700 °C for 1 h in air.

In addition, control sample of Ta_2_O_5_ (99.5 wt %) powders was purchased from Shanghai Chemicals Corporation for control experiment.

### Characterization of Ta-doped ZnO Nanoparticles

The structure of the particles was characterized by a powder X-ray diffraction (XRD) equipment (D/max2000, Rigaku) using Cu Ka radiation. The Ta-doping content was determined by inductively coupled plasma resonance (ICP, JY 38S, JY). The morphology and microstructure of nanoparticles were examined by scanning electron microscope (SEM, Philips XL-30, The Netherlands) and transmission electron microscope (TEM, Tecnai G^2^ F20, Philips). The surface chemical composition of the nanoparticles were investigated by X-ray photoelectron spectroscopy (XPS, Thermo Fisher K-Alpha) with standard Al Kα (1486.7 eV) X-ray source. The binding energy scale was calibrated using the energy position of the adventitious C 1*s* peak at 284.6 eV.

### Bacterial Strains and Culture Conditions

For antibacterial experiments, Gram-positive *B. subtilis* and *S. aureus* and Gram-negative *E. coli* and *P. Aeruginosa* were four etiological agents of several infective diseases in humans, chosen as the target organism. *E. coli* (ATCC25922), *S. aureus* (ATCC6538), *B. subtilis* (CMCC63501), and *P. aeruginosa* (ATCC 27853) were purchased from Guangzhou Microbiology Research Center. All tubes and materials were sterilized in an autoclave before the experiments. Nutrient broth was used to culture *E. coli*, *S. aureus*, *B. subtilis*, and *P. aeruginosa* at 37 °C for 2 days on a rotary platform in an incubator. The liquid cultures were finally diluted to obtain bacterial cell concentration of approximately 10^7^ colony-forming units (CFU)/ml for following antibacterial test.

### Evaluation of Antibacterial Activity

The MIC of Ta-doped ZnO nanoparticles was measured by broth dilution test in dark condition. Phosphate buffered saline (PBS, 2.5 ml) and broth solution (2.5 ml) were added to the tubes containing the 10^7^ CFU inoculation. Then, Ta-doped ZnO nanoparticles with various amounts of 100–1000 μg/ml were added into the tubes. The negative control tube did not contain any inoculum, and positive control tube was free of antibacterial agent. The tubes were incubated at 37 °C for 48 h on a rotary platform. The visual turbidity of the tubes was noted before and after incubation. The MIC was defined as the endpoint where no visible turbidity could be detected.

The antibacterial activity of Ta-doped ZnO nanoparticles was also evaluated under visible light illumination. The tube samples containing various bacteria were prepared as above. Based on the MIC results, the concentration of antibacterial agent suspensions in tubes was set to 160 μg/ml for *B. subtilis* and 100–200 μg/ml for *E. coli*, *S. aureus*, and *P. aeruginosa*, respectively. A 300-W Xe arc lamp (300W, Ushio) was located 15 cm away from the tubes, providing the visible light above 425 nm through a cut-off filter. After visible light irradiation for various durations of 0–240 min, the tubes were cultured for at 37 °C on a rotary platform. The cultivation time in an incubator was equal to the irradiation time. The growth curve was determined by measuring the time evolution of the optical density (OD) of the sample contained in a 10-mm optical path length quartz cuvette. The density of bacterial cells in the liquid cultures was estimated by OD measurements at 600 nm wavelength using a UV-vis-NIR spectrophotometer (UV-3600, Shimadzu) at a frequency of once every 20 min.

In order to make a comparative study, pure ZnO and Ta_2_O_5_ powders were also used as control samples for antibacterial test under visible light and dark ambient. The control tubes were prepared and incubated under the similar conditions.

To investigate the antibacterial behavior of Ta-doped ZnO nanoparticles, SEM were also used to examine the morphology of some bacteria samples with and without antimicrobial treatment under visible light and dark ambient.

## Results and Discussion

### Characterization of Ta-doped ZnO Nanoparticles

**Fig. 1:**
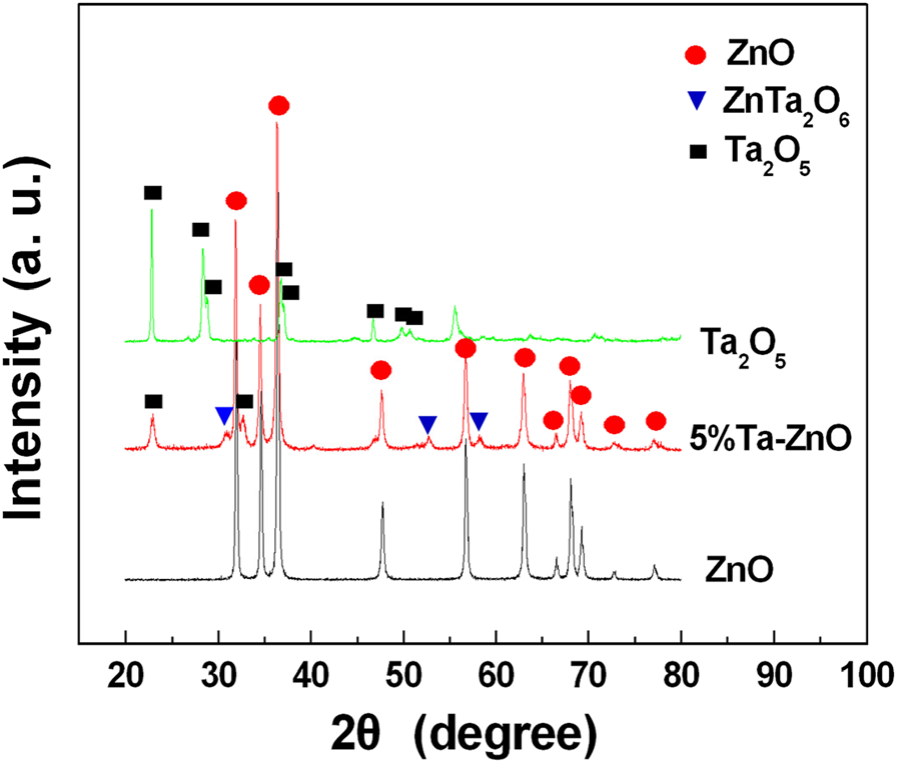
XRD patterns of pure ZnO, Ta-doped ZnO nanoparticles, and Ta_2_O_5_ powders

Figure 1 shows the XRD patterns of pure ZnO and 5 % Ta-doped ZnO nanoparticles annealed at 700 °C. One and 3 % Ta-doped ZnO samples show pure hexagonal wurtzite structure of polycrystalline ZnO (JCPDS card No.16-1451) with slightly increasing *a* and *c* lattice constant (not shown here), indicating that the larger Ta^5+^ ions occupy the Zn^2+^ sites to form Ta-doped ZnO solid solution [27]. When Ta-doping content is 5 %, an evident peak at 23.1° and 32.6° from Ta_2_O_5_ can be observed, and meanwhile, some weak peaks at 30.2°, 52.8°, and 58.3° can be indexed to secondary phase of orthorhombic ZnTa_2_O_6_ (JCPDS card 39-1484). For comparison, the XRD pattern of commercial Ta_2_O_5_ powders is also given in Fig. 1 with orthorhombic phase (JCPDS card No.71-0639). The crystallite sizes are estimated from the Scherer formula to be around 38, 30, and 53 nm, corresponding to pure ZnO, 5 % Ta-doped ZnO, and Ta_2_O_5_ powders, respectively.

**Fig. 2:**
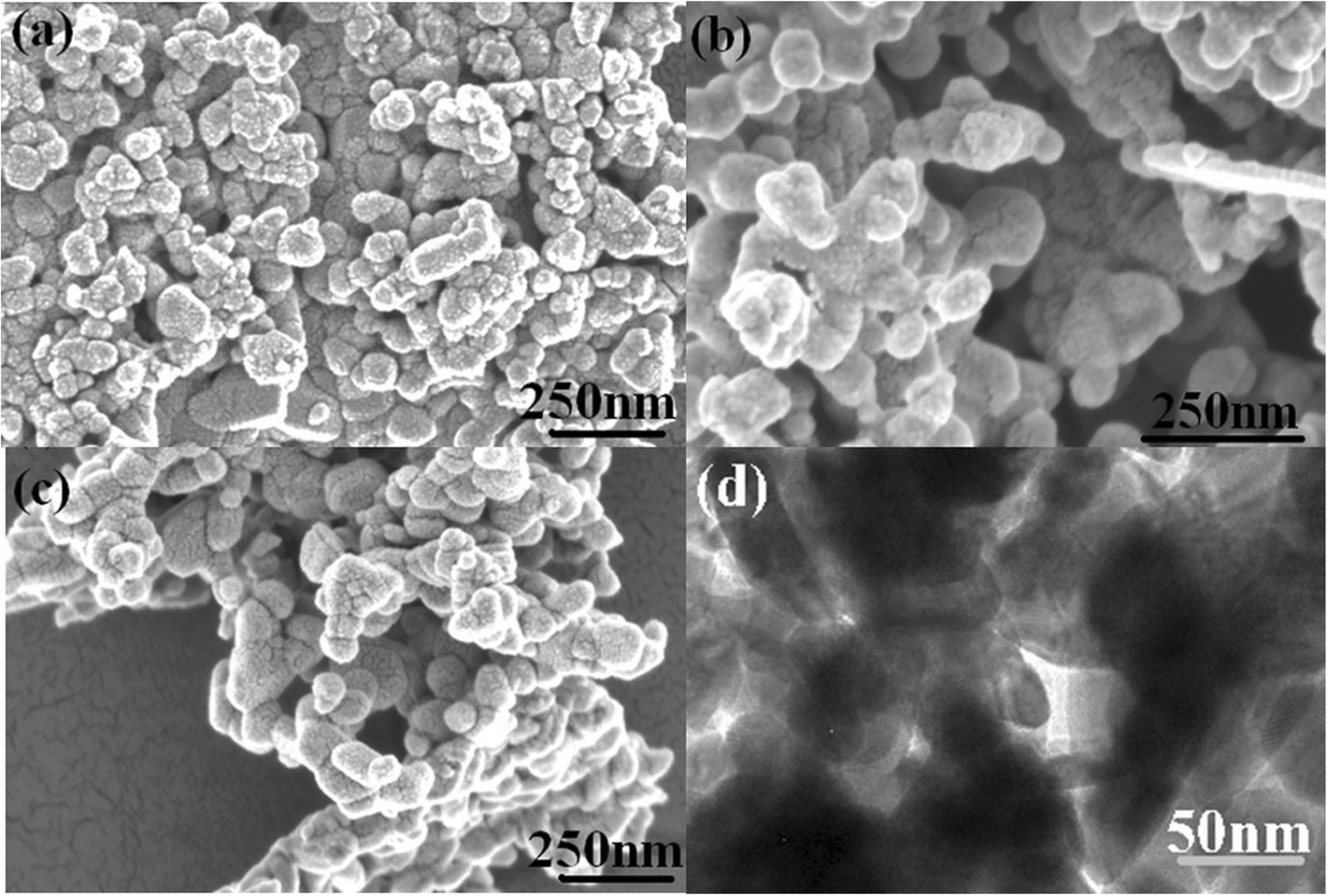
SEM and TEM images of various powders. **a** pure ZnO. **b** 5 % Ta-doped ZnO nanoparticles. **c** Ta_2_O_5_ powders. **d** 5 % Ta-doped ZnO nanoparticles (TEM image)

Typical SEM images of pure ZnO, 5 % Ta-doped ZnO nanoparticles, and Ta_2_O_5_ powders are shown in Fig. 2a–c. Figure 2d displays a TEM image of 5 % Ta-doped ZnO nanoparticles. Pure ZnO and Ta-doped ZnO nanoparticles are approximately spherical shaped with the dispersive diameters of 10–60 nm. These smaller nanoparticles easily assemble together to form some clusters of several hundred nanometers. Commercial Ta_2_O_5_ powders exhibit smaller grain of ~40–60 nm. A HRTEM image (not shown here) of 5 % Ta-doped ZnO nanoparticle records the disorder regions and some dislocations in the surface layer and grain boundary. Therefore, it can be deduced that there exist more defects on the surface of the 5 % Ta-doped ZnO nanocrystals by a modified PC method.

### Antibacterial Test Results

MIC was determined after incubation at 37 °C for 48 h in dark ambient by the standard microbial method, as summarized in Table 1. It is easily seen that all samples including ZnO, Ta-doped ZnO, and Ta_2_O_5_ exhibit better biocidal effect on *B. subtilis* and worse antibacterial activity on *P. aeruginosa* bacteria. Meanwhile, with increasing the Ta content to 3–5 %, the growth of *E. coli* and *S. aureus* is effectively inhibited. For *E. coli* and *S. aureus*, no significant antibacterial activity is observed for pure ZnO, 1 % Ta-doped ZnO, and Ta_2_O_5_ samples at maximum concentration of 1000 μg/ml. The lowest MIC for *E. coli*, *S. aureus*, and *B. subtilis* is 180, 200, and 160 μg/ml after adding the 5 % Ta-doped nanoparticles into the tubes, respectively. When continuing to increase the Ta-doping amount from 5 to 10 % in ZnO, the antibacterial effect is similar. The MIC results indicate that the Ta-doping ion can improve the antibacterial effect of ZnO nanoparticles on *E. coli*, *S. aureus*, and *B. subtilis* under dark conditions.

**Table 1:** MIC results of ZnO and Ta-doped ZnO with various Ta contents (unit: μg/ml)

Bacteria	ZnO	1 % Ta-doped	3 % Ta-doped	5 % Ta-doped	Ta_2_O_5_
*P. aeruginosa*	N.D.	N.D.	N.D.	N.D.	N.D.
*E. coli*	N.D.	N.D.	200–250	180	N.D.
*S. aureus*	N.D.	N.D.	500	200	N.D.
*B. subtilis*	350–400	230–240	200	160	200

*N.D.* not determined (it means that no antibacterial effect is observed at the concentration of 1000 μg/ml)

Many bacteriological tests have shown that ZnO suspensions in the lower concentration range (0.01–1 mM, i.e., 0.8–80 μg/ml) exhibit less antimicrobial activity [31, 32]. Our MIC results also confirm this point. Fewer Zn^2+^ ions might act as nutrient-a supplement promoting the metabolic action of bacteria at trace concentrations. So, usually, ZnO are believed to be nontoxic, biosafe, and biocompatible and have been used in many applications in daily life, such as drug carriers, in cosmetics, and fillings in medical materials.

**Fig. 3:**
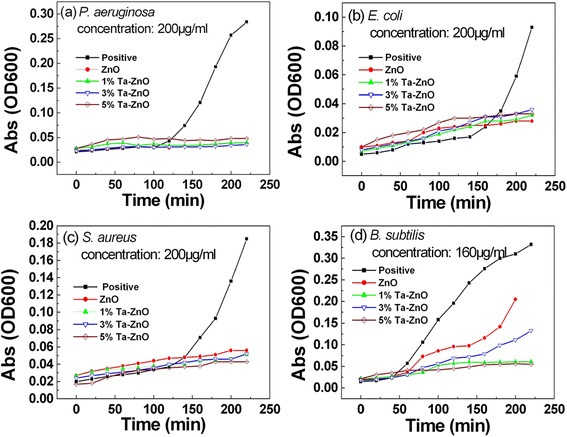
Growth curves of bacteria with time evolution in the presence of Ta-doped ZnO nanoparticles with different Ta contents under visible light. **a** *P. aeruginosa.* **b** *E. coli.* **c** *S. aureus.* **d** *B. subtilis*

The antibacterial activity of Ta-doped ZnO nanoparticles was also evaluated under visible light illumination. Spectrophotometry were explored to assess bacterial growth because spectrophotometric readings are generally more precise and reproducible [33–35]. Figure 3 shows the growth curves of four bacteria with time evolution in the presence of Ta-doped ZnO nanoparticles with different Ta contents under visible light. All samples of Ta-doped ZnO and pure ZnO nanoparticles with 100–200 μg/ml concentration of antibacterial agent exhibit evident antibacterial effect on *P. aeruginosa*, *E. coli*, and *S. aureus* under visible light irradiation in Fig. 3a–c; however, quite a few of these samples such as pure ZnO and 1 % Ta-doped ZnO nanoparticles do not show antibacterial activity in dark conditions, even if at 1000 μg/ml concentration. Moreover, the biocidal effect on *P. aeruginosa* is quite different under visible light and dark ambient. The visible light irradiation greatly improves antibacterial activity of *P. aeruginosa*.

**Fig. 4:**
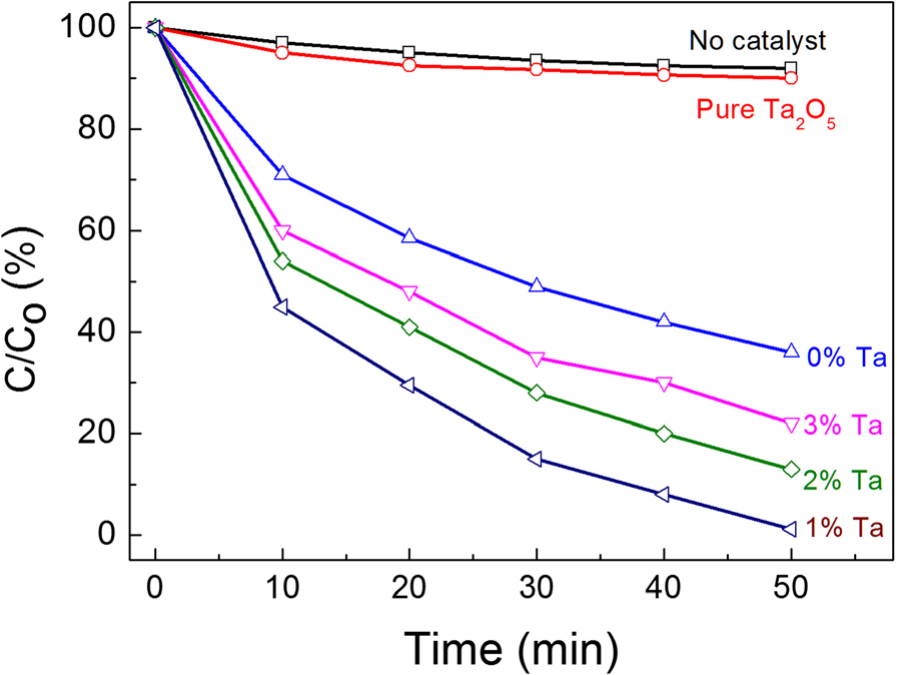
Visible photodegradation of methylene blue with Ta-doped ZnO, pure ZnO, and Ta_2_O_5_ powders

At present, for *P. aeruginosa*, *E. coli*, and *S. aureus*, Ta-doped ZnO nanoparticles show almost the same antibacterial activity as ZnO under visible light illumination. Our previous work on visible photodegradation of methylene blue with Ta-doped ZnO nanocrystals indicates that pure ZnO nanoparticles with defects also exhibits photocatalytical activity under visible light [27, 28], as indicated in Fig. 4. Maybe, the present exposure dose of antimicrobial agent is not low enough to distinguish the antibacterial activity between Ta-doped ZnO and ZnO nanoparticles.

In addition, compared to MIC result under dark conditions, the visible light irradiation has a hardly significant influence on antibacterial activity of *B. subtilis* at 160 μg/ml concentration (see Fig. 3d). For samples containing pure ZnO and 3 % Ta-doped ZnO nanoparticles, the gradually enhanced OD with time indicates the worse microbial control on *B. subtilis*, similar to the previous MIC results. Five percent Ta-doped ZnO nanoparticles show also similar antibacterial activity of *B. subtilis* under visible light and in the absence of light. The only exception is that 1 % Ta-doped ZnO nanoparticles exhibit slightly enhanced biocidal effect on *B. subtilis.* Maybe it is related to the fact that 1 % Ta-doped ZnO nanoparticles has optimal photocatalytical activity in Fig. 4 [28].

**Fig. 5:**
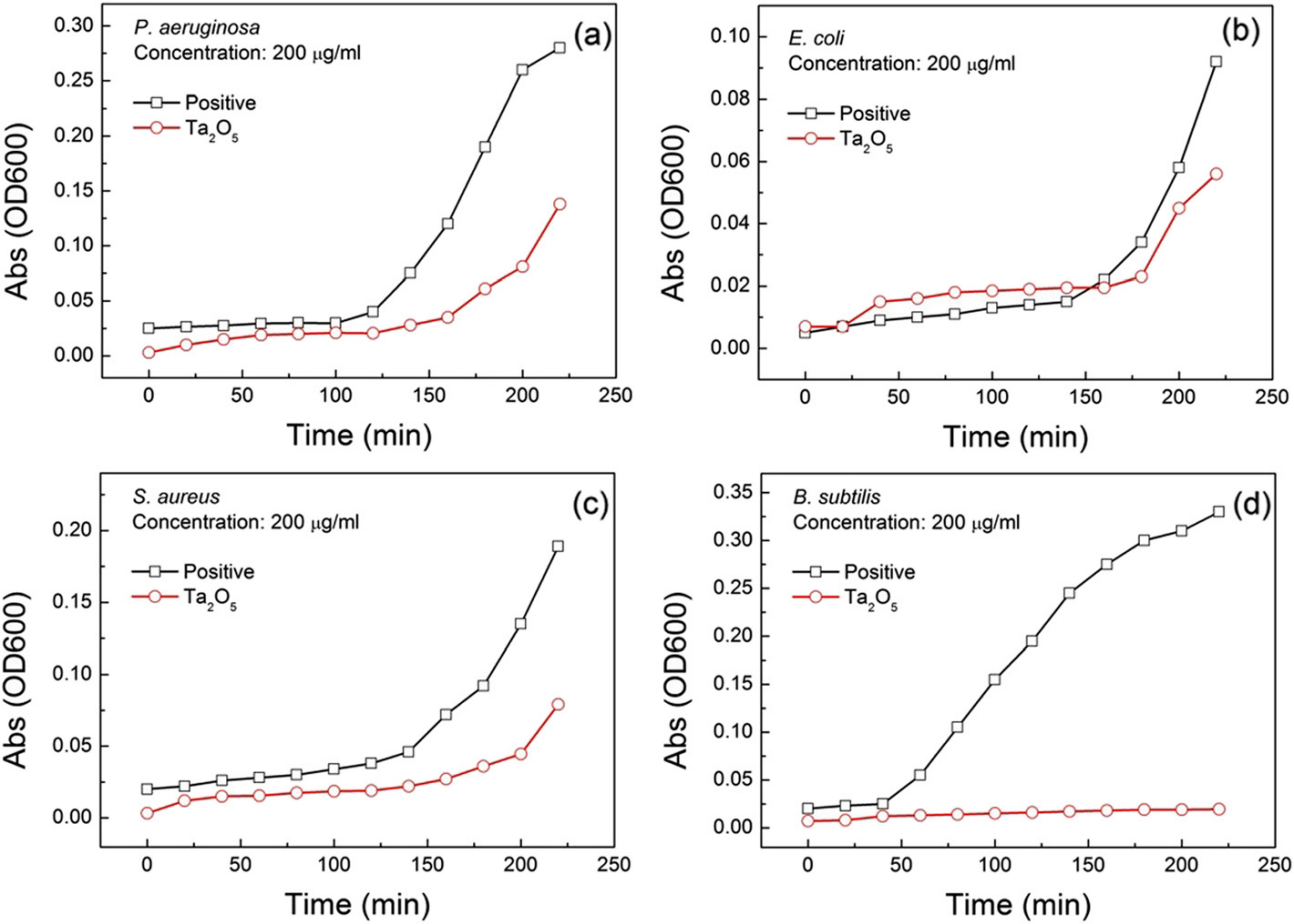
Growth curves of bacteria with time evolution in the presence of Ta_2_O_5_ powders under visible light. **a** *P. aeruginosa.* **b** *E. coli.* **c** *S. aureus.* **d** *B. subtilis*

In order to understand the Ta role in the photocatalyst, the similar photocatalysis test of Ta_2_O_5_ powders at 200 μg/ml concentration under visible light was also performed, as seen in Fig. 5a–d. It is clearly seen that Ta_2_O_5_ powders have evident bactericidal efficacy on *B. subtilis* under visible light illumination (Fig. 5d), similar to the MIC results in the absence of light. Compared to positive controls, Ta_2_O_5_ powders show weak antibacterial effect on *P. aeruginosa*, *E. coli*, and *S. aureus* under visible light irradiation, as seen in Fig. 5a–c. The rapid increase of OD value after 200 min indicates that Ta_2_O_5_ powders do not effectively prevent *P. aeruginosa*, *E. coli*, and *S. aureus* from multiplying. Based on the photodegradation of methylene blue with Ta_2_O_5_ powders under visible light (Fig. 4), there is hardly a photocatalytical activity for Ta_2_O_5_ powders. Why Ta_2_O_5_ powders exhibit better biocidal action on only *B. subtilis* in dark ambient or light irradiation is unclear now. Maybe, it is related to certain special role of Ta^5+^ ion.

## Discussion on the Antibacterial Mechanisms

**Fig. 6:**
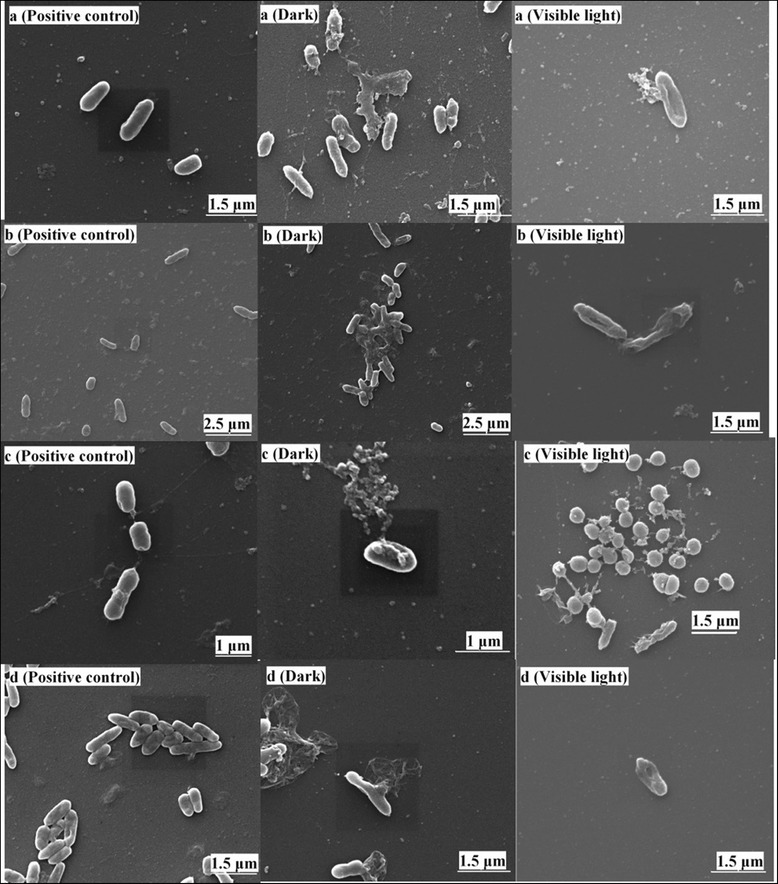
SEM images of four kinds of bacteria with and without anti-treatments. **a** *P. aeruginosa* positive control and treated with 200 μg/ml of 3 % Ta-ZnO by MIC under dark ambient and with 160 μg/ml of 1 % Ta-ZnO under visible light for 10 min. **b** *E. coli* positive control and treated with 300 μg/ml of 3 % Ta-ZnO by MIC under dark ambient and with 160 μg/ml of 1 % Ta-ZnO under visible light for 10 min. **c** *S. aureus* positive control and treated with 200 μg/ml of 1 % Ta-ZnO by MIC under dark ambient and with 160 μg/ml of 1 % Ta-ZnO under visible light for 10 min. **d** *B. subtilis* positive control and treated with 160 μg/ml of 1 % Ta-ZnO by MIC under dark ambient and with 160 μg/ml of 1 % Ta-ZnO under visible light for 10 min

To gain direct evidence of the antibacterial behavior of Ta-doped ZnO nanoparticles, SEM were used to characterize the bacterial morphology with and without anti-treatments. Figure 5 shows the four kinds of bacteria images of positive control samples, samples treated with Ta-doped ZnO nanoparticles under dark and visible light ambient. The difference of bacteria morphology between the positive control and anti-treated samples is easily observed. The former keeps better cell shape and cell state; however, the latter shows torn cell membrane, destruct tissue, and large areas of dead bacteria. The whole cell death process can be seen from Fig. 6. First the membrane hole appears, then the cell tissue outflows, and finally the bacteria are killed. It seems that the damage to the cell membrane directly leads to the leakage of minerals, proteins, and genetic materials, causing cell death.

Although the anti-treatments of the bacteria with Ta-doped ZnO nanoparticles under dark and visible light ambient lead to considerable damage to cell membranes, compared to the MIC under dark ambient, the antibacterial effect is significantly enhanced in visible light. The anti-treatment on *P. aeruginosa*, *E. coli*, *S. aureus*, and *B. subtilis* for 10 min under visible light illumination by photocatalysis produces similar injury effect to that for 48 h in the absence of light by MIC in Fig. 6. Evidently, the antibacterial mechanism of ZnO and Ta-doped ZnO under visible light is different from under dark ambient.

ZnO powders have been studied as inorganic antimicrobial agents due to their photocatalytic activity under UV light. Some mechanisms have been proposed. The exact mechanism of antibacterial activity of ZnO is still unclear and may involve generation of ROS, release of Zn^2+^ ions, and damage to cell membranes [19]. Tunney et al. thought that the photocatalytic generation of H_2_O_2_ might be one of the primary mechanisms [35]. The suspension of ZnO nanoparticles with defects can be activated by both UV and visible light and produce electron-hole pairs (e^−^h^+^). The holes split H_2_O molecules into OH^−^ and H^+^. Dissolved oxygen molecules react with e^−^ and H^+^ in turn, and finally, molecules of H_2_O_2_ are obtained. For Ta-doped ZnO nanoparticles, similar reactions also exist under visible light [36].

1 $$ \mathrm{T}\mathrm{a}\hbox{-} \mathrm{Z}\mathrm{n}\mathrm{O} + \mathrm{h}\upnu \to \kern0.5em \mathrm{T}\mathrm{a}\hbox{-} \mathrm{Z}\mathrm{n}\mathrm{O}\ \left({\mathrm{e}}^{-} + {\mathrm{h}}^{+}\right) $$

2 $$ {\mathrm{h}}^{+}\kern0.5em +\kern0.75em {\mathrm{H}}_2\mathrm{O}\to \kern0.5em \cdot \mathrm{O}\mathrm{H}\kern0.5em +\kern0.5em {\mathrm{H}}^{+} $$

3 $$ {\mathrm{e}}^{-}\kern0.5em +\kern0.5em {\mathrm{O}}_2\kern0.5em \to \kern0.5em \cdot {{\mathrm{O}}_2}^{-} $$

4 $$ \cdot {{\mathrm{O}}_2}^{-}\kern0.5em +\kern0.5em {\mathrm{H}}^{+}\to \kern0.5em {\mathrm{H}\mathrm{O}}_2\cdot $$

5 $$ {\mathrm{H}\mathrm{O}}_2\cdot \kern0.5em +\kern0.5em {\mathrm{H}}^{+}\kern0.5em +\kern0.5em {\mathrm{e}}^{-}\to \kern0.5em {\mathrm{H}}_2{\mathrm{O}}_2 $$

Under visible light irradiation, an electron-hole pair forms, and then a conduction-band electron and a valence-band hole separate on the surface of Ta-doped ZnO (Eq. 1). The high oxidative potential of the holes can form very reactive hydroxyl groups through decomposing of water (Eq. 2). In the presence of the dissolved O_2_, electrons from the photoexcited Ta-doped ZnO produce superoxide anion radicals ·O_2_^−^ (Eq. 3), which subsequently could generate H_2_O_2_ by the intermediate HO^2−^ and HO_2_· steps (Eqs. 4, 5). The free radicals ·O_2_^−^ and ·OH produced in the reactions can react with organic substance inside bacterial cells to produce bacterial toxins, leading to death of the bacteria. Especially, the generated H_2_O_2_ easily penetrates into the cell membrane and kills the bacteria [37].

H_2_O_2_ produced by visible light catalytic process might play an important role in antibacterial test, so we measured the H_2_O_2_ content of Ta-doped ZnO, pure ZnO, and Ta_2_O_5_ solution under visible light (concentration of 200 μg/ml) at 0 and 40 min, as shown in Table 2. H_2_O_2_ has been detected in all samples, even if in solution containing Ta_2_O_5_ powders. Obviously 1 % Ta-doped ZnO produces the highest H_2_O_2_ content and Ta_2_O_5_ with lowest H_2_O_2_ content after 40 min visible light irradiation; however, it is difficult to establish the clear relationship between antibacterial activity and generated H_2_O_2_ under visible light because except Ta_2_O_5_, pure ZnO and Ta-doped ZnO with 1, 3, and 5 % contents exhibit better antibacterial activity on *P. aeruginosa*, *E. coli*, and *S. aureus* under visible light illumination. Although now we cannot determine which factor is the most important in antibacterial mechanism, without doubt the photocatalytic activity of Ta-doped ZnO and pure ZnO under visible light plays a key role.

**Table 2:** The H_2_O_2_ content of different samples of 200 μg/ml at 0 and 40 min under visible light

Samples (μg/ml)	ZnO	1 % Ta	3 % Ta	5 % Ta	Ta_2_O_5_
0 min	28.7	86.3	64.7	64.7	14.3
40 min	39.4	100.7	50.3	43.1	21.5

Recently, Yin group reported the photogenerated charge carriers and reactive oxygen species in ZnO/Au hybrid nanostructures with enhanced photocatalytic and antibacterial activity by electron spin resonance (ESR) spectroscopy [19]. Maybe next, we may try ESR measurements to determine the generation of ROS and photoinduced charge carriers (electron or hole) of Ta-doped ZnO under visible light so as to better understand the antibacterial mechanism and clarify the correlation between charge carrier formation, generation of reactive intermediates, and antibacterial activity, especially, the role of individual ROS in antibacterial activity of photoexcited Ta-doped ZnO.

Our previous work shows the introduction of Ta^5+^ ions into ZnO can cause a series of changes in structure, morphology, and photocatalytical degradation of methylene blue, such as larger lattice parameter, smaller grain size, and more active defect sites and hydroxyl groups [28, 29]. Based on the antimicrobial study on Ta-doped ZnO nanoparticles, the incorporation of Ta^5+^ ions indeed improves the biological activity in dark ambient in most cases. The MIC results show that except the *P. aeruginosa* samples, the Ta-doping significantly enhances the bacteriostasis effect of ZnO nanoparticles on *E. coli*, *S. aureus*, and *B. subtilis* in the absence of light. Usually, ZnO particles directly adsorbed to the cell walls may inhibit bacterial growth by penetration of the cell envelope and disorganization of bacterial membrane. The cell wall rupture is related to the surface activity of ZnO in contact with the bacteria.

Ta^5+^ as a high valence metal ion doped into ZnO lattice may generate zinc vacancy or oxygen interstitial in order to keep the electric neutral equilibrium, leading to the deviation of O/Zn ratio from stoichiometry. Table 3 lists the XPS results of pure ZnO and 3 % Ta-doped ZnO before and after Ar-ion etching. The Ar-ion etching was utilized to remove the surface adsorption oxygen of nanoparticles. The O/Zn ratio of 1.20 for 3 % Ta-doped ZnO is higher than 0.97 for pure ZnO after Ar-ion etching, implying that the introduction of Ta^5+^ ions leads to the formation of oxygen interstitial defect in 3 % Ta-doped ZnO. Evidently, the Ta-doped ZnO undoubtedly contains more defects with reduced grain size, improving the surface activity of ZnO. On the other hand, Stoimenov et al. ever reported that the electrostatic forces may strength the binding of the metal oxide nanoparticles on the bacteria surface, facilitating the bactericidal action [38]. The incorporation of high valence Ta^5+^ ion into ZnO lattice also enlarges the electrostatic force, producing stronger adsorption and binding of nanoparticles on bacteria. Ta-doped ZnO nanoparticles exhibit more effective bactericidal efficacy than pure ZnO in dark ambient, which can be attributed to the synergistic effect of enhanced surface bioactivity and increased electrostatic force due to the incorporation of Ta^5+^ ions into ZnO.

**Table 3:** The XPS results of ZnO and 3 % Ta-doped ZnO before and after Ar-ion etching

Samples	Ar-ion etching	O	Zn	O/Zn ratio
ZnO	No	44.09	32.01	1.38
ZnO	Yes	45.52	46.96	0.97
3 % Ta-doped ZnO	No	37.94	28.04	1.35
3 % Ta-doped ZnO	Yes	50.43	42.06	1.20

Additionally, for various bacteria, the antibacterial action of Ta-doped ZnO nanoparticles under visible light and dark ambient shows some differences. Under dark environment, Ta-doped ZnO nanoparticles exhibit very weak bacteriostasis effect on *P. aeruginosa*, but in visible light irradiation, strong bactericidal efficacy on *P. aeruginosa* is observed. For *B. subtilis*, the action of the photocatalysis of Ta-doped ZnO seems to be limited. These phenomena might be related to various structures and functions of several bacteria. Further in-depth work is needed.

Above all, according to our antibacterial tests, 5 % Ta-doped ZnO is a more effective antimicrobial agent than pure ZnO.

## Conclusions

In summary, novel photocatalyst of Ta-doped ZnO nanoparticles was prepared by a modified Pechini-type method. The antibacterial activity of Ta-doped ZnO nanoparticles on several bacteria of *P. aeruginosa*, *S. aureus*, *E. coli*, and *B. subtilis* were investigated using a standard microbial method. The Ta-doping concentration effect on MIC of various bacteria under dark ambient has been evaluated. The photocatalytical biocidal behavior of Ta-doped ZnO nanoparticles under visible light irradiation was also characterized. The MIC results indicate that the incorporation of Ta^5+^ ions into ZnO significantly improve the bacteriostasis effect of ZnO nanoparticles on *E. coli*, *S. aureus*, and *B. subtilis* in the absence of light. Compared to MIC results without light irradiation, Ta-doped ZnO and pure ZnO nanoparticles show much stronger bactericidal efficacy on *P. aeruginosa*, *E. coli*, and *S. aureus* under visible light illumination. The possible antibacterial mechanisms in Ta-doped ZnO systems under visible light and dark conditions have been proposed. Ta-doped ZnO nanoparticles exhibit more effective bactericidal efficacy than pure ZnO in dark ambient, which can be attributed to the synergistic effect of enhanced surface bioactivity and increased electrostatic force due to the incorporation of Ta^5+^ ions into ZnO. Based the antibacterial tests, 5 % Ta-doped ZnO is a more effective antimicrobial agent than pure ZnO.
